# UniBind: a novel artificial intelligence-based prediction model for SARS-CoV-2 infectivity and variant evolution

**DOI:** 10.1038/s41392-023-01691-2

**Published:** 2023-12-19

**Authors:** Qihong Yan, Jincun Zhao

**Affiliations:** State Key Laboratory of Respiratory Disease, National Clinical Research Center for Respiratory Disease, Guangzhou Institute of Respiratory Health, the First Affiliated Hospital of Guangzhou Medical University, Guangzhou, China

**Keywords:** Predictive medicine, Bioinformatics

In a recent study published in *Nature Medicine*, Wang et al. developed an excellent framework called UniBind based on artificial intelligence (AI), which enables accurately predicting infectivity of SARS-CoV-2 variants and evolutionary trends of future viral variants.^1^ This computational method holds the possibility to not only serve as a valuable early-warning tool for monitoring potential pathogenic SARS-CoV-2 variants but also facilitate fundamental research on protein-protein interactions (PPIs).

PPIs between the virus and the host protein form the foundation of host-virus interactions and virus evolution. Understanding how virus mutation affects PPIs is critical for prediction of the genesis and evolution of newly emerged SARS-CoV-2 variants. Recently, several bioinformatic approaches have been developed to predict the impacts of binding affinities on SARS-CoV-2 receptor binding domain (RBD) mutations. However, those computational approaches are limited by their performance and/or throughput on newly emerged viral variants, highlighting the urgent need to build an advanced artificial intelligence system.

**Fig. 1:**
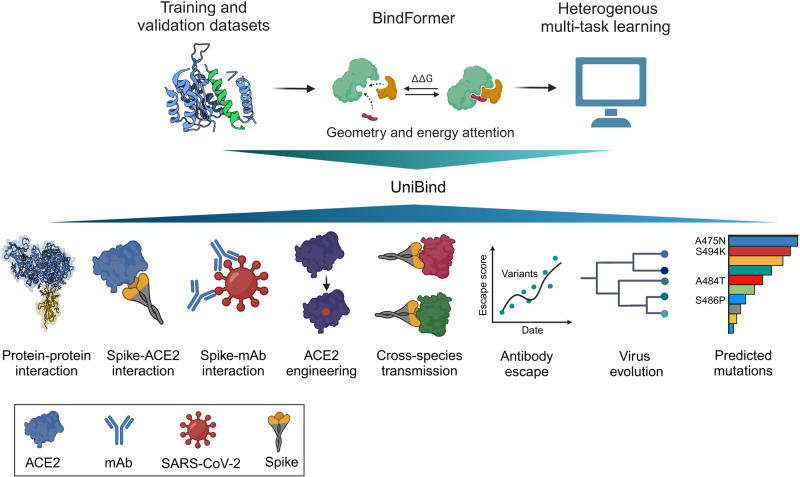
Workflow and functional properties of UniBind framework. The UniBind consists of three major components. After trained using heterogeneous multitask learning methods for PPI prediction tasks, UniBind was applied to conduct multiple prospective predictions and exhibited great performance. Monoclonal antibody: mAb. Created by BioRender.com

This study by Wang et al. proposed a deep learning framework called UniBind by integrating heterogeneous biological data from available databases such as SKEMPI, MaveDB, IGBPG, and GISAID. These comprehensive data resources provide promising opportunities for AI model training and validation. The UniBind framework contains three main components: (1) protein represented as multiple scale; (2) BindFormer modules with Geometry & energy attention (GEA); (3) heterogeneous multi-task learning. Briefly, affinity data collected from public databases were interpreted and then inputted into the BindFormer block. Next, The BindFormer block exchanged information within multi-scale representations through GEA. Then, multi-task learning was applied for model training and internal validation. Furthermore, deep mutational scanning (DMS) data from MaveDB and other experiments were used for external validation of the performance and reliability of UniBind. Lastly, the well-trained UniBind model was applied to conduct prospective analyses such as AI-based DMS, AI-based lineage analysis, model-guided evolution, and viral fitness landscape evaluation (Fig. 1).

UniBind was trained and verified employing the latest database, SKEMPI V.2.0, to predict the changes of mutations on protein–protein interactions. Overall, the AI prediction results are accurate, with Pearson’s correlation coefficient (PCC) of 0.85. UniBind exhibits great prediction performance regardless of whether the protein–protein complexes contained single or multiple mutations, with PCCs of 0.78 and 0.91, respectively. Furthermore, the authors used the MaveDB and IGBPG databases to independently verify the performance of UniBind, and achieve ideal PCC values, suggesting that UniBind can predict binding affinity effectively and the prediction was highly correlated with experimental binding data.

After the comprehensive training and validation, UniBind was robust for evaluating the binding affinity between mutated Spike and ACE2, with PCCs ranging from 0.78 to 0.86 for four different SARS-CoV-2 variants. Similarly, UniBind was also accurate for predicting affinity between Spike and neutralizing antibodies, and all the PCCs were higher than 0.8 for four major classes of RBD-targeting antibodies. By generating escape scores of pandemic variants to representative neutralizing antibodies, predictions of UniBind showed that the Omicron lineage display the strongest immune escape capacity, consistent with previous published literatures.

In addition to enable prediction of the affinities between Spike and ACE2 mutations, UniBind was able to generate ACE2 variants which possess increased affinities with Spike, highlighting that Unbind can serve as a potential tool for engineering therapeutic ACE2 proteins. Next, Wang et al., extended their predictions on the cross-species binding affinities between ACE2 and RBD, achieving PCC of 0.87. Furthermore, UniBind was used to predict the impact of substitutions in SARS-CoV-2 variants on ACE2 binding affinities of twenty-four animal species. This analysis revealed that except for pigs, all the other twenty-three animal species are easily infected with SARS-CoV-2 variants. Notably, Omicron lineage has shown an enhanced binding ability to all animal species, suggesting the possible risk of cross-species transmission of SARS-CoV-2.

Given that UniBind was capable of predicting binding affinity and antibody escape at single-mutation level accurately, Wang et al. further used UniBind to estimate neutralizing antibody and ACE2 binding affinities of the pandemic SARS-CoV-2 variants that carried multiple mutations, as well as depict their emergence during the course of COVID-19 pandemic. This prediction revealed an overall enhancement in immune escape. Based on the hypothesis that SARS-CoV-2 evolved toward enhanced immune escape or/and ACE2 binding capacity, an evolutionary score system (evo-score) based on binding affinity was generated to estimate both Spike-antibody and Spike-ACE2 binding affinity and tested their performance employing the GISAID database. On the one hand, the predominant enhancement of Spike–ACE2 binding affinity led to the evolution of SARS-CoV-2. On the other hand, the accumulation of substitutions in the Spike is consistent with the increasing capacity in antibody escape, which is likely driven by herd immunity established by infection or/and vaccination.^2,3^

To facilitate the advanced development of vaccines and targeted therapeutics before the emergence of new variants, UniBind was prospectively applied to predict the evolution trend of SARS-CoV-2. Notably, the AI model successfully predicted the emergence of E484T and F486P substitutions, which were observed so far in recently dominant variants XBB.1.3 and XBB.1.5. In addition, the prediction also demonstrated that S494K and A475N substitutions appear to have strong immune evasion capabilities and may be emerged in future SARS-CoV-2 variants, which deserve close attention and surveillance.

Overall, UniBind serves as an excellent AI framework for estimating the affinity of virus–host protein complex and shining light on accurate monitoring of future outbreaks and rapid development of effective therapeutics and vaccines. In addition, two other AI-based models namely DML^4^ and MLAEP^5^ were also developed recently, both of which exhibit great performance for PPI predicting. The UniBind together with DML and MLAEP are useful resources for research and public health management such as coronavirus, influenza, HIV, and monkeypox virus pandemics.
